# A New Functional Classification of Glucuronoyl Esterases by Peptide Pattern Recognition

**DOI:** 10.3389/fmicb.2017.00309

**Published:** 2017-02-28

**Authors:** Jane W. Agger, Peter K. Busk, Bo Pilgaard, Anne S. Meyer, Lene Lange

**Affiliations:** Center for BioProcess Engineering, Department of Chemical and Biochemical Engineering, Technical University of DenmarkLyngby, Denmark

**Keywords:** glucuronoyl esterase, PPR, CE15, lignin carbohydrate complexes, glucuronoxylan

## Abstract

Glucuronoyl esterases are a novel type of enzymes believed to catalyze the hydrolysis of ester linkages between lignin and glucuronoxylan in lignocellulosic biomass, linkages known as lignin carbohydrate complexes. These complexes contribute to the recalcitrance of lignocellulose. Glucuronoyl esterases are a part of the microbial machinery for lignocellulose degradation and coupling their role to the occurrence of lignin carbohydrate complexes in biomass is a desired research goal. Glucuronoyl esterases have been assigned to CAZymes family 15 of carbohydrate esterases, but only few examples of characterized enzymes exist and the exact activity is still uncertain. Here peptide pattern recognition is used as a bioinformatic tool to identify and group new CE15 proteins that are likely to have glucuronoyl esterase activity. 1024 CE15-like sequences were drawn from GenBank and grouped into 24 groups. Phylogenetic analysis of these groups made it possible to pinpoint groups of putative fungal and bacterial glucuronoyl esterases and their sequence variation. Moreover, a number of groups included previously undescribed CE15-like sequences that are distinct from the glucuronoyl esterases and may possibly have different esterase activity. Hence, the CE15 family is likely to comprise other enzyme functions than glucuronoyl esterase alone. Gene annotation in a variety of fungal and bacterial microorganisms showed that coprophilic fungi are rich and diverse sources of CE15 proteins. Combined with the lifestyle and habitat of coprophilic fungi, they are predicted to be excellent candidates for finding new glucuronoyl esterase genes.

## Introduction

Glucuronoyl esterases are classified as CAZymes family 15 carbohydrate esterases. Currently, glucuronoyl esterases receive considerable attention due to their proposed activity toward ester-linkages in the intermolecular connection points between lignin and carbohydrates in lignocellulosic biomass (Špániková and Biely, 2006). Covalent linkages between lignin and carbohydrates are collectively called lignin-carbohydrate-complexes (LCCs) and consist of several linkage types (Balakshin et al., 2011) depending on the source of biomass. In glucuronoxylan-rich types of biomass (e.g., hardwood) LCCs consist of ester linkages formed between α-1,2 linked 4-*O*-methyl-glucuronoyl substitutions on xylan and the aliphatic chains of lignin (either α- or γ positioned) (Li and Helm, 1995). The common notion is that hydrolysis of LCCs will relieve some of the constraints believed to limit impact of enzymatic hydrolysis of lignocellulose and not only increase the yield of carbohydrates but also the purity of lignin (Biely, 2016).

The CE15s in the CAZy-database^1^ currently consists of eight characterized proteins. Most of these eight proteins (six out of eight) have not been assigned with a full EC-number yet owing to the undecided esterase activity. The remaining two bacterial proteins have been assigned respectively to EC.3.1.1.72 (acetyl xylan esterase and is also found in CE3) and to EC.3.2.1.40 (α-L-rhamnosidase, also found in GH78). The bacterial CE3 protein (from *Ruminococcus flavefaciens*) also hydrolyses the ester-linkage in a synthetized 4-*O*-methyl-D-glucuronic acid esterified to methanol (Biely et al., 2015). This dual substrate specificity might be explained by the multimodular nature of the protein (Aurilia et al., 2000). CE15 esterases are an underexplored family of enzymes and like many of the other CAZy-families, the CE15s may harbor more than one type of esterase activity.

Glucuronoyl esterase activity was first discovered by coincidence (Špániková and Biely, 2006) and isolated from a culture broth of the Basidiomycetous fungus *Schizophyllum commune* (Agaricales). The enzyme is active toward methyl ester of 4-*O*-methyl-D-glucuronic acid and has no activity toward any of the otherwise common carbohydrate esterase substrates. It was later reported that the same enzyme was active against other synthetic arylalkyl alcohol esters of both 4-*O*-methyl-D-glucuronic acid and D-glucuronic acid (Špániková et al., 2007). Kinetic data clearly indicate that the presence of a 4-*O*-methyl group on glucuronic acid highly increases the hydrolytic activity of the enzymes and that the glucuronoyl moiety is more important for substrate recognition than the aromatic group (Špániková et al., 2007; d’Errico et al., 2015).

Interestingly, the authors (Špániková and Biely, 2006) suggested that the *S. commune* esterase is not a serine type esterases due to lack of inhibition by common serine-protease inhibitor PMSF. Later crystal structures and point mutations of glucuronoyl esterases from the Ascomycetous fungi *Hypocrea jecorina* (Hypocreales) (Pokkuluri et al., 2011) and *Myceliophthora thermophila* (Sordariales) (Topakas et al., 2010; Charavgi et al., 2013) have finally confirmed the presence of a classical Ser-His-Glu catalytic triad; this is now believed to be the molecular motif responsible for the catalytic activity.

Up until now, a variety of different synthetic substrates has been used to study the activity of glucuronoyl esterases (Katsimpouras et al., 2014; d’Errico et al., 2015; Nylander et al., 2015; Sunner et al., 2015) besides the methyl ester of 4-*O*-methyl-D-glucuronic acid. Very recently, glucuronoyl esterase activity on naturally derived substrates was suggested by Arnling Bååth et al. (2016) and d’Errico et al. (2016) but there is still no understanding of how the glucuronoyl esterases function together with other carbohydrate acting enzymes in lignocellulose deconstruction. Providing clear, direct proof of glucuronoyl esterase activity on natural substrate and interactions with other enzymes is a challenging task but also a most crucial step to expand the understanding of the biological role of these enzymes. We hypothesized that a first prerequisite to attain an improved understanding of these enzymes would be to provide an overview and more importantly to pin-point possible function similarities and differences among the proteins classified as CE15 and CE15-like.

In this study, we used peptide pattern recognition (PPR) to identify putative CE15 enzymes. PPR is a relatively new sequence analysis technology and software platform for non-alignment-based identification of conserved motifs in related protein sequences (Busk and Lange, 2013). PPR finds a limited number of highly conserved n-mer peptides among input protein sequences. When the function of the sequences is known, e.g., as is the case for the characterized proteins in the CAZy-database^1^ PPR can be used to find proteins with similar sequence features and predict their function. For this study we pooled the CE15 proteins listed in CAZy with fungal, plant and bacterial CE15-like proteins found in GenBank. A total of 1024 unique proteins were analyzed with PPR to generate 24 groups of related CE15 proteins belonging to the expanded CE15 protein family. Moreover, genomic screening indicated a higher number of CE15-encoding genes in lignin degrading coprophiles and white rot fungi than in brown rot fungi. Furthermore, a number of putative CE15-encoding genes were found in lignocellulolytic bacteria.

## Materials and Methods

### Sequence Analysis

Amino acid sequences of all 115 CE15 proteins in CAZy (Lombard et al., 2010) were downloaded from GenBank in November 2015. The sequences were classified with PPR as previously described with the parameters peptide length = 6, conserved peptides per protein = 10 and number of conserved peptides per group = 70 (Busk and Lange, 2013).

To generate expanded protein families the top hit in each of the four PPR-generated CE15 groups and a selection of the unclassified sequences were used for BLAST search (Altschul et al., 1997) in GenBank. The 1000 top hits for each search were pooled and duplicates were removed. Next, protein domains in the sequences were mapped with dbCAN (Yin et al., 2012) and domains clearly not related to CE15, and not overlapping with a CE15 domain were deleted. All sequences shorter than 51 amino acids after deletion of unrelated domains were removed.

The 1024 curated protein sequences were analyzed by PPR and the proteins in each PPR group were analyzed with dbCAN.

Pairwise sequence alignment was made with MUSCLE (Edgar, 2004) and adjusted manually. Phylogenetic trees were made with MUSCLE, PhyML and TreeDyn at www.phylogeny.fr (Marseille-Nice genopole^®^, Centre National de la Recherche Scientifique, Réseau National des Génopole) (Dereeper et al., 2008).

All analyses were performed on online servers.

### Distribution of Hexapeptides in the Proteins

The position of a conserved hexapeptide was defined as the median of the position in all the protein sequences that contained the hexapeptide sequence. Using the median instead of the average position compensates for the different length of the sequences and for the occurrence of truncated proteins.

### Gene Annotation

All predicted proteins in the genome-sequenced bacteria and fungi as indicated in Supplementary Table S1 were downloaded from GenBank. Each protein was given a score for each group-specific PPR-determined peptide list as previously described (Busk and Lange, 2015) and annotated to the PPR group with the highest score.

Furthermore, ORFs were generated from the genomes of the fungi *Armillaria fuscipes* (Agaricales*), Ganoderma lucidum* and *Wolfiporia cocos* (both Polyporales) as previously described (Busk and Lange, 2015). Briefly, genomes were downloaded and split into fragments of 2000 bases with 100 bases overlap. The fragments were translated in all six reading frames and all reading frames longer than 50 residues were given a score for each group-specific peptide list as previously described (Busk and Lange, 2015).

## Results and Discussion

**FIGURE 1:**
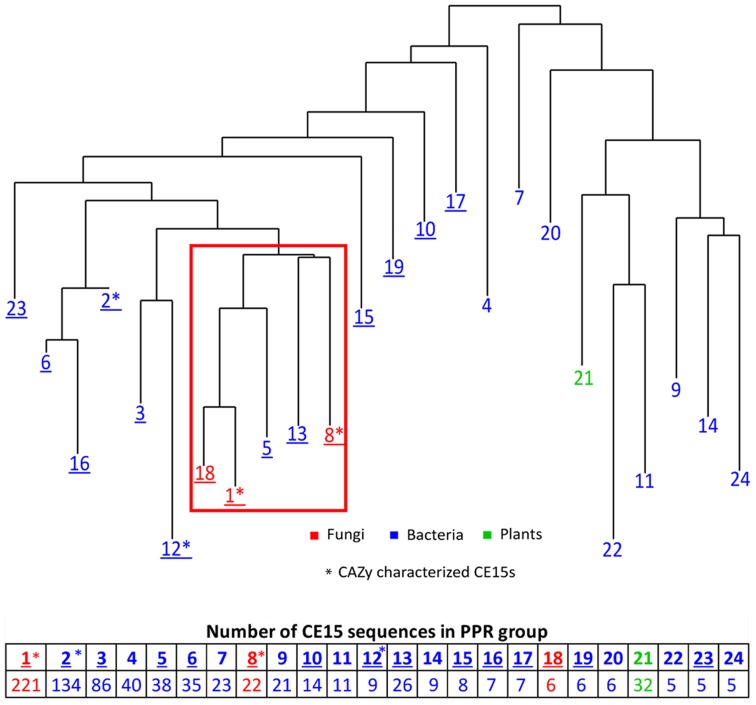
**Phylogenetic clustering of the organisms harboring the top one ranking CE15 protein sequence in 24 unique PPR groups**. Table below shows the number of CE15 protein sequences in each PPR group. Red color indicate PPR groups exclusively or almost exclusively composed of CE15 sequences with fungal origin, blue color indicate PPR groups where the CE15 sequences are of bacterial origin and green color indicate one PPR group consisting of CE15 sequences of plant origin. Asterisks (^∗^) indicates in which PPR groups the CAZy characterized CE15s fall. Groups with CE15 proteins already annotated in CAZy are marked by underlining of the PPR group number.

Peptide pattern recognition was used to divide the 115 CE15 protein sequences found in CAZy into four groups of proteins. To identify more CE15 proteins we used these groups to find in all 1024 CE15-like protein sequences in GenBank. PPR analysis divided these proteins into 24 groups of proteins with similar, conserved peptides. All fungal CE15 proteins were found in groups 1, 8, and 18 whereas the other 21 PPR groups contained bacterial sequences and one PPR group (21) contained sequences from plants. Phylogenetic analysis of the sequence with the highest score in each PPR group (**Figure 1**) showed that groups 1, 8 and 18, containing the fungal sequences, cluster closely together. Interestingly, the bacterial CE15 sequences from groups 5 and 13 were closely related to the fungal sequences from groups 1, 8, and 18. PPR groups 1 and 8 contain six out of the eight glucuronoyl esterases currently described in CAZy. Pairwise comparison showed that the identity between the highest scoring sequences in PPR groups 1, 5, 8, 13, and 18 (marked by a red square in **Figure 1**) ranges from 30 to 56%. Such high sequence identity suggests that these CE15 enzymes have the same or very similar functional activity. The close relation of PPR groups 5, 8, 13, and 18 to PPR group 1 is supported by the finding that these groups share 1–5 conserved peptides with PPR group 1 (Supplementary Figure S1).

Peptide pattern recognition group 1 contains by far the most putative CE15 protein sequences and also most of the already described glucuronoyl esterases. Proteins that fall into PPR group 18 all originate from one organism (*Coprinopsis cinerea*, Agaricales) and cluster closely together with PPR group 1; e.g., they form one sub-branch in a phylogenetic tree including the sequences of PPR group 1 and PPR group 18 (**Figure 1**). Moreover, each of these protein sequences was at least 71% identical to a sequence from PPR group 1. While it is interesting that one organism has unique structural properties among its CE15 protein sequences, it adds little information to the overall function prediction of CE15 sequences, and PPR group 18 may be considered functionally identical to PPR group 1. Thus it will be treated as such in the text below.

**FIGURE 2:**
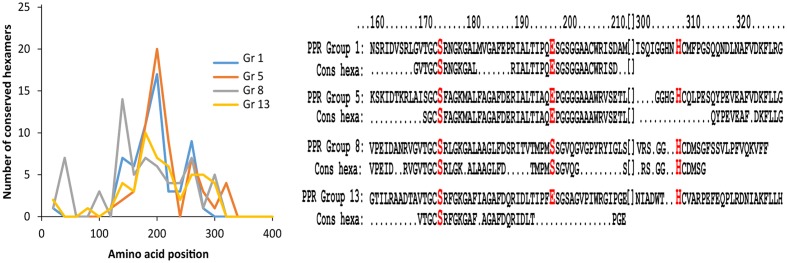
**Conserved hexamers in PPR groups 1, 5, 8, and 13**. Left hand diagram showing the number of conserved hexamers in PPR groups 1, 5, 8, and 13. The x-axis displays the position of the conserved hexamers on the entire protein sequence, and the y-axis displays the number of conserved hexamers. PPR groups 1, 5, and 13 show the highest number of conserved hexamers in the range from 160 to 200 amino acids within the protein sequence. PPR group 8 shows highest region of conservation around position 140. All groups also share a conserved motif around position 260. Right hand diagram shows alignment of protein sequence around the catalytic triad with conserved peptides from amino acid 160 to amino acid 320 of the top scoring protein sequence in PPR group 1 (Protein XP_001834719.1), 5 (Protein WP_044269389.1), 8 (Protein XP_003352714.1), and 13 (Protein WP_037992621.1). Below each protein sequence is listed the conserved hexapeptide (cons hexa) designated to the individual PPR group. The alignments show high conservation around the catalytic serine and glutamic acid residues, except the protein sequence from PPR group 8 which has another amino acid (S) in the glutamic acid position. The catalytic histidine is positioned in a region of less conservation.

The annotated CE15 sequences in CAZy are classified in the PPR groups 1–3, 5, 6, 8, 10, 12, 13, 15–19, and 23 on the left hand side of the phylogenetic tree (**Figure 1**, underlined). CDD search identifies many of the protein sequences in the remaining groups (right hand side of the phylogenetic tree) as members of the α/β-hydrolase superfamily of hydrolytic enzymes. In agreement with this finding, 71 of the 152 proteins in these families are designated as hydrolytic enzymes in GenBank (**Table 1**). The most frequently predicted activities are sialidases (26 proteins in two groups) and acetyl xylan esterases (16 proteins distributed in four PPR groups). However, BLAST search with the sequences predicted to be sialidases found both putative sialidases and acetyl xylan esterases. Sialidases have a six-bladed β propeller topology (Amaya et al., 2004). This is different from the structure of α/β-hydrolases (8 β-strands connected by 6 α-helices). Moreover, the α/β-hydrolases are characterized by a highly conserved catalytic triad with a catalytic serine residue (Ollis et al., 1992). This catalytic serine is also found in PPR groups 1, 5, 8, and 13 (**Figure 2**). By aligning the highest scoring protein in PPR groups 1 and 5 to the sequences in PPR groups 7, 9, 11, 14, 20, 21, 22, and 24 it was possible to identify the motif around a putative catalytic serine. Based on the identification of the putative catalytic serine and the α/β-hydrolase fold of the sequences it is reasonable to suggest that the proteins on the right hand side of the phylogenetic tree (**Figure 1**) are carbohydrate esterases that may be active on acetylated xylan or similar substrate. This finding is in agreement with the identification by PPR, that these sequences are closely related to the CE15 esterases annotated in CAZy. The presence of putative sialidase activity agrees well with the fact that also sequences from non-lignocellulosic plant species are represented in the right hand side of the phylogenetic tree in group 21 (**Figure 1**).

**Table 1:** GenBank description of uncharacterized sequences and identification of the catalytic serine.

Group	Description from GenBank	Catalytic serine motif
4	Twenty four sialidases	GHSF/GHSY
7	Six acetylxylan esterases Eight peptidases Seven dipeptidyl aminopeptidases	GHSL
9	Four acetylxylan esterases One Peptidase S15 domain protein	GRSG
11	One hydrolase	GMSM
14	Five acetylxylan esterases	GCSG/GESG
20	Two sialidases One dienelactone hydrolase-like enzyme	GHSL
21	Four putative hydrolases YtaP Four putative esterases YitV	GISL/GMSL
22	Two acetylxylan esterase Two dienelactone hydrolases	GISG
24	One metalloendopeptidase-like membrane protein	GNSG/GISG

Recently, a CE15 protein was identified from a metagenomics search of marine bacteria most likely from a *Ruminococcus flavefaciens* (De Santi et al., 2016). While this protein falls into the CE15 family, it has low activity toward glucuronoyl-ester but relatively high activity toward various acetylated substrates. In addition to the activity differences between this new CE15 and the previously described glucuronoyl esterases, also structural differences related to the catalytic triad is suggested, namely that the catalytic acid is something else than glutamic acid and that major structural differences in the tertiary structure occurs. PPR places this protein in PPR group 12, a PPR group only consisting of proteins originating from *Ruminococcus flavefaciens* and this group is closely related to PPR group 3. This new protein proves that other esterase activities than glucuronoyl esterases are found in the CE15 family of enzymes.

A closer examination of PPR groups 1, 5, 8, and 13 shows that the region of conservation is largest from 160 to 200 amino acids (**Figure 2**). An alignment of the top ranking protein sequences with the conserved hexameric peptides in each of these PPR groups reveals a large agreement with the catalytic residues serine and glutamic acid in the region around position 170 except for group 8, which has a serine residue instead of the glutamic acid. All proteins in PPR group 8 have a serine in this position and since PPR group 8 proteins also differed in region of mostly conserved hexapeptides (**Figure 2**) a likely different evolution for PPR group 8 proteins may have occurred compared to the other PPR groups in the cluster. The region around the catalytic histidine is somewhat less conserved compared to the serine and glutamic acid regions.

The conserved peptides for the expanded CE15 search were used to screen the genomes of a selection of known cellulose and lignocellulose degrading fungi and bacteria [coprophilic fungi, white rot fungi, gray rot fungi (as a sort of fungi with lifestyles resembling both white rot and brown rot (Riley et al., 2014), brown rot fungi and lignocellulose degrading bacteria]. The purpose of doing this was to identify groups of organisms, belonging to such physiologically specialized growth forms, which had the highest prevalence of CE15 protein sequences hence revealing where to look for the best enzyme candidate genes for finding CE15 proteins with the desired glucuronoyl esterase activity. Such screening should be followed by cloning, expression and characterization to confirm the hypothesis. The entire list of organisms and the results of the screening can be found in Supplementary Tables S2–S6 and Figure S1.

**FIGURE 3:**
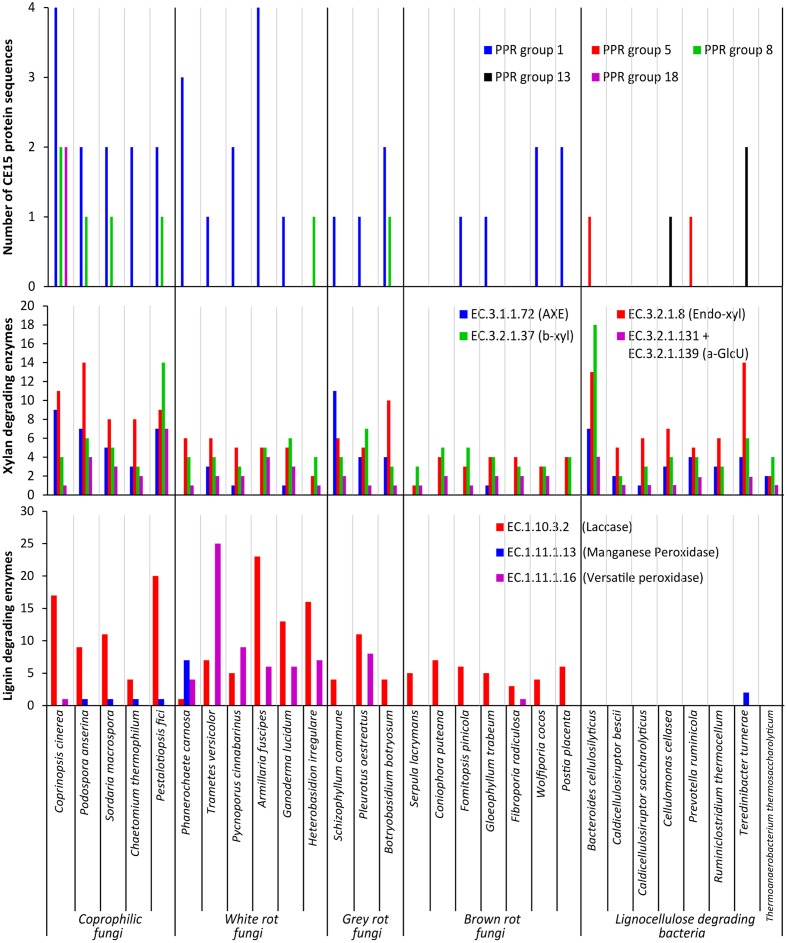
**Functional annotation of CAZymes in selected fungal and bacterial organisms**. Results shown as a summary of their individual CE15 proteins divided by PPR group **(Top)**, their glucuronoxylan degrading activities **(Middle)** defined as acetyl xylan esterase (AXE EC 3.1.1.72), endo-β-1,4-xylanase (Endo-xyl EC. 3.2.1.8), β-xylosidase (β-xyl EC 3.2.1.37) and α-glucuronidase (a-GlcU EC 3.2.1.131 + EC 3.2.1.139) and lignin degrading activities **(Bottom)** defined as laccase EC 1.10.3.2, manganese peroxidase EC 1.11.1.13 and versatile peroxidase EC 1.11.1.16.

The average number of CE15 protein sequences in each kind of organisms (**Table 2**) revealed that the highest number of CE15-encoding genes was found in coprophilic fungi; second highest found in white rot fungi; gray rot fungi as number three; and brown rot fungi and bacteria had the lowest average number of CE15 genes in their genomes. The identified CE15 proteins primarily belong to PPR group 1 (**Figure 3**, top diagram) and the diversity of CE15 proteins is highest among coprophilic fungi, where all three fungal PPR groups (1, 8, and 18) were found (**Figure 3**).

**Table 2:** Average number of protein sequences in each type of fungal organisms; coprophiles, white rot, gray rot, and brown rot respectively and lignocellulose degrading bacteria.

	# enzymes, CE15	# enzymes, glucuronoxylan degradation	# enzymes, lignin degradation
Coprophilic fungi	4,0	77,6	16,0
White rot fungi	2,0	39,3	26,2
Gray rot fungi	1,7	58,3	10,0
Brown rot fungi	0,9	28,1	6,6
Lignocellulose degrading bacteria	0,6	43,1	0,3

*# enzymes, CE15 indicates the number of CE15 proteins identified by PPR; # enzymes, glucuronoxylan degradation indicates the average number of the sum of CAZy family proteins related to degradation of glucuronoxylan defined as acetyl xylan esterases (CE1-6 + CE12), endo-1,4- β-xylanase (GH10-11 + GH30), β-xylosidase (GH3 + GH43) and α-glucuronidase (GH67 + GH115). # enzymes, Lignin degradation indicates the average number of the sum of CAZy family proteins associated with lignin degradation defined as laccase (AA1) and peroxidases (AA2). The CAZy families mentioned here are prevalent families for the activities in question, however they may also contain other enzyme activities*.

A taxonomic approach to the results illustrated in **Figure 3** give an additional aspect of where the different types of CE15 genes are found: the highest CE15 scoring genome among both coprophilic and white rot fungi are the Basidiomycetous species belonging to the Agaricales (*Coprinopsis cinerea* and *Armillaria fusciopes*). All the four Ascomycetous species among the selected Coprophilic species have the same CE15 profile: PPR group 1 (two genes) and 8 (one gene). The lowest scoring of the white rots are the two species belonging to the Russulales *T. versicolor* (PPR group 1) and *H. irregulare* (PPR group 8). Among the gray rots, the two species belonging to Agaricales have one gene of PPR group 1; different from *B. botryosum* which has PPR group 1 (two genes) and PPR group 8 (one gene). None of the taxonomic groupings among the Brown rots scored high. Two of the three species with nil CE15 belonged to the Basidiomycetous Boletales, however one of the Polyporales species among the brown rots *F. radiculosa* also had nil CE15.

Interestingly PPR groups 5 and 13 encoding genes were only found in the bacterial genomes; the overall impression is that CE15 proteins are less common in bacteria, despite the fact that the organisms selected here are known to be lignocellulose degraders in nature.

Because glucuronoyl esterases are believed to function on LCCs particularly formed by linkage between glucuronoxylan and lignin we found it interesting to correlate the occurrence of CE15 proteins to the general capacity of glucuronoxylan and lignin degradation. On average about 80% of the proteins annotated to a CAZy family are also functionally annotated (Busk et al., 2014). The average number of glucuronoxylan degrading enzymes [defined as EC 3.1.1.72 (acetyl xylan esterase, represented by the sum of CAZy family enzymes CE1-6 + CE12), EC 3.2.1.8 (endo-1,4-β-xylanase, represented by the sum of CAZy family enzymes GH10, GH11, and GH30), EC 3.2.1.37 (β-xylosidase, represented by the sum of CAZy family enzymes GH3 and GH43) and the sum of 3.2.1.131 and 3.2.1.139 (α-glucuronidase, represented by the sum of CAZy family enzymes GH67 and GH115)] is highest in coprophiles (**Table 2**), but the gray rot fungal organisms are also surprisingly efficient in turnover of glucuronoxylan as they have an average of 58 genes for xylan degradation. Again, the brown rot organisms seem to be the least efficient enzyme producers for glucuronoxylan degradation. The most widespread enzymes with confirmed activity for xylan degradation is the endo-xylanase activity (EC 3.2.1.8) (**Figure 3**, middle diagram) with an average of 10 proteins among the coprophiles, five for white rot, seven for gray rot, and three for brown rot. Also bacterial microorganisms are rich in endo-xylanases with an average of 7. Surprisingly, the α-glucuronidase activity (EC 3.2.1.131 and EC 3.2.1.139) is in all fungal organisms mostly represented by the GH115 family of enzymes (Supplementary Table S2) rather than by the GH67 family. Only some of the coprophilic fungi and the bacterial species harbor GH67 genes. GH67 α-glucuronidases are known for their exclusive activity on non-reducing end positioned glucuronoyl substituents, whereas GH115 α-glucuronidases act on endo-positioned substitutions. A plausible combined action of α-glucuronidase and glucuronoyl esterase could be initial release of longer xylan-oligosaccharides from the LCC linkage by the α-glucuronidase followed by release of 4-*O*-methyl-glucuronic acid by the glucuronoyl esterase to complete the yield of carbohydrates from lignin. Acetyl xylan esterase activity, which is found in the CAZy family’s CE1–CE6 and CE12 is normally also related to xylan degradation. In agreement with this notion the white rot fungi genomes contain an average of 13 CE-encoding genes, dominated by CE1 and CE4 families. Interestingly, only few of these genes are closely related to the characterized EC 3.1.1.72. This finding suggests that a large part of the sequence variation of acetyl xylan esterases can be elucidated by further characterization of CE15 family enzymes from the white rot and the coprophilic fungi.

Enzymatic lignin degradation is traditionally linked to the presence of peroxidases (manganese and versatile peroxidases) and laccases (Fernandez-Fueyo et al., 2012), and coprophilic and white rot microorganisms are particularly rich in enzymes with these activities (**Figure 3**, lower diagram). The classical definition of the basidiomycetous groupings white and brown rot is exactly linked to the content of enzymes acting on lignin, and it is therefore not surprising that the brown rot fungi selected for this study show low occurrence of lignin degrading enzymes. Also the bacteria selected here seem based on genomic data rather inefficient in enzymatic lignin conversion. It is a known fact that brown rot fungi do not convert lignin for metabolic purposes; whether this is also the case for brown rot like bacteria or if bacteria have developed other enzymatic mechanisms for breaking down and utilizing lignin is not known. Observations in nature suggest, however, that lignin remnants after break down of the lignocellulosic substrate by brown rot fungi can remain intact for long periods of time and apparently are not easily broken down by bacteria. The term brown rot fungi were originally coined based on the finding of such brown remnants of the wood structure.

The coprophilic fungal species seem to be those with the most versatile set of enzymes for attacking complex substrates containing LCCs. The ecological habitat of coprophilic fungi is the droppings of herbivorous animals where they pass the gastro-intestinal tract before proliferation in the dung. This specialized life form growing on highly processed biomass requires enzymes that can cope with the most difficult parts of plant cell walls. This makes coprophilic fungi an attractive source for discovery of enzymes with specialized functions for degrading also recalcitrant parts of the lignocellulose structure.

## Conclusion

By employing PPR analysis of protein sequences from a high number of fungal and bacterial organisms we have constructed an expanded CE15 family and identified and divided putative CE15 proteins into groups based on their sequence similarity. Phylogenetic analysis of these CE15 proteins reveals that fungal CE15 proteins together with some bacterial proteins seem to have a high degree of sequence conservation and suggests a similar activity (PPR groups 1, 3, 5, 8, 13, and 18). At the same time, we identified a number of previously unnoticed CE15-like proteins with putative esterase activity and α/β-hydrolase structure. The low sequence identity to known proteins suggests that the CE15 family of enzymes could hold several types of activities and not exclusively glucuronoyl esterases. Coupling the occurrence of CE15 proteins to the overall capacity of enzymatic lignocellulose degradation among coprophilic fungi and white, gray and brown rot fungi reveal the coprophilic fungal species to be the richest source of putative glucuronoyl esterases; richest both in number of different CE15 genes and in diversity with regard to being representing highest number of different CE15 PPR groups. Furthermore, white rot fungi are also shown to have highly interesting and still uncharacterized CE15 enzymes. The highest diversity of CE15 genes in coprophilic and white rot fungi are found among the Basidiomycetous species belonging to the order Agaricales.

## Author Contributions

JA, PB, and LL all contributed substantially to the conception of the hypotheses and the design of the work. PB, BP, and JA performed the data acquisition, analysis and figure design. JA, PB, BP, AM, and LL all contributed to the data interpretation, preparation and final approval of the manuscript.

## Conflict of Interest Statement

The authors declare that the research was conducted in the absence of any commercial or financial relationships that could be construed as a potential conflict of interest. The reviewer AMR and handling Editor declared their shared affiliation, and the handling Editor states that the process nevertheless met the standards of a fair and objective review.

## Footnotes

1 http://www.cazy.org
