# Supplementary material for: A New Functional Classification of Glucuronoyl Esterases by Peptide Pattern Recognition
Source: Front Microbiol. 2017 Feb 28;8:309. doi: 10.3389/fmicb.2017.00309 (PMC5329029; doi:10.3389/fmicb.2017.00309)
Supplement: Supplementary file 7 [file Data_Sheet_1.DOCX]

**Figure S1: Protein accession numbers and conserved peptides in PPR groups**

PPR Group 1

Accession numbers: XP_001834719.1;EQB54590.1;XP_007286128.1;XP_007598828.1;KFY13447.1;KFY29710.1;KFY84204.1;KIM21095.1;KFY75708.1;KFZ07425.1;KKY22299.1;XP_003654509.1;KFX90335.1;KFX97800.1;XP_007337783.1;EKG16633.1;KFZ10809.1;KIJ48607.1;KJA29997.1;XP_003345667.1;KIJ25069.1;XP_011114526.1;EGZ69793.1;KFY09204.1;KIM21134.1;XP_009852891.1;XP_007841831.1;KIY66140.1;XP_007912900.1;KNG52590.1;XP_001834720.1;XP_007585921.1;EUN23454.1;XP_007715365.1;XP_007697190.1;ERT00520.1;KIH91046.1;XP_007684439.1;XP_014082256.1;KJR84976.1;XP_001931471.1;XP_007844565.1;KNZ75990.1;KFY57954.1;XP_003844265.1;XP_008095957.1;XP_003346801.1;XP_007798936.1;XP_007337788.1;GAP84434.1;XP_011123978.1;XP_003306006.1;XP_008023029.1;XP_008033523.1;XP_001799602.1;XP_008087696.1;KDR66406.1;KFH40442.1;XP_007847217.1;XP_958597.2;KDN60408.1;XP_009222849.1;ELQ41644.1;KOP46181.1;KDQ19074.1;4G4G;CCA74892.1;XP_001227750.1;KDQ23481.1;KLU91213.1;XP_001905727.1;KIJ35374.1;ENH89185.1;KEQ65864.1;XP_008084971.1;KEQ80432.1;XP_006692313.1;WP_051235210.1;XP_001586190.1;KKY35379.1;KDQ11090.1;EEB91637.1;XP_007677514.1;XP_007330841.1;AIY68500.1;XP_006455315.1;KOP42405.1;XP_001226041.1;WP_012486493.1;KFY28243.1;WP_024461787.1;XP_013427163.1;AIF91552.1;KJA21004.1;CAN91155.1;XP_013314287.1;WP_049876171.1;CDO70357.1;XP_007759899.1;WP_052705079.1;XP_013328797.1;WP_052691889.1;KIK69580.1;XP_003026289.1;4G4I;XP_007366803.1;ACE84239.1;EJT98565.1;KIO32983.1;CRG87022.1;CRK43806.1;KEY71968.1;KFA50635.1;KIV83559.1;KFA81775.1;KFA67599.1;XP_006695403.1;XP_007340692.1;CRK11666.1;XP_001834722.2;KIJ48602.1;WP_014546979.1;XP_013937545.1;XP_008724944.1;XP_013960044.1;EQB51796.1;XP_007281900.1;KIJ56173.1;KIN02722.1;3PIC;KIW65987.1;XP_006969119.1;XP_002471858.1;WP_016777553.1;XP_001903136.1;KKO96622.1;XP_007868664.1;KIO32982.1;KIJ59676.1;KIK59793.1;WP_056587031.1;XP_007390689.1;KOP45051.1;WP_051718199.1;EDP55347.1;EMD31275.1;WP_052691782.1;XP_007837912.1;KIP12537.1;EMD32859.1;KIM97588.1;KMK63723.1;XP_751313.1;WP_027752106.1;KIJ38575.1;XP_001258567.1;WP_050753050.1;WP_012234846.1;WP_019708157.1;WP_014313110.1;WP_034629037.1;WP_043466128.1;KGM02342.1;WP_030544142.1;WP_020816983.1;WP_024833960.1;KLO19318.1;XP_007792309.1;WP_033340689.1;XP_007387720.1;WP_055478457.1;ADI11428.1;WP_018837155.1;WP_027769492.1;WP_027743075.1;WP_023361630.1;XP_001211031.1;GAO88533.1;WP_034270975.1;WP_043487920.1;WP_018843396.1;WP_027774889.1;WP_037742644.1;WP_018851477.1;WP_051704594.1;WP_045826296.1;WP_028885850.1;CCF42078.1;WP_026193546.1;WP_028876450.1;ADG89472.1;XP_001832002.2;KNZ82096.1;WP_028883284.1;KLO13335.1;WP_030509784.1;WP_019604187.1;WP_019602103.1;WP_026932111.1;WP_026225164.1;XP_001832002.2;KDN19124.1;WP_026923997.1;KIJ49145.1;WP_020511007.1;WP_049579717.1;XP_002559165.1;KII95650.1;BAL91158.1;WP_052705216.1;WP_053715194.1;WP_037082742.1;KIJ62413.1;WP_041590269.1;AFN75319.1;XP_001841601.1;XP_007303694.1;WP_051772107.1;WP_050753051.1;WP_018014222.1;WP_043181753.1

Conserved peptides: VTGCSR,0.79;RNGKGA,0.63;SRNGKG,0.63;GVTGCS,0.63;GCSRNG,0.61;CSRNGK,0.61;AWAWGV,0.61;TGCSRN,0.53;ENVWFS,0.52;ESGSGG,0.52;QESGSG,0.51;VAPRGL,0.49;IPQESG,0.48;LTIPQE,0.48;TIPQES,0.47;PQESGS,0.47;AWGVSR,0.44;RIALTI,0.43;WAWGVS,0.43;WGVSRI,0.43;TAWAWG,0.41;VSRIID,0.41;IALTIP,0.4;ALTIPQ,0.4;GVSRII,0.38;IDWLGP,0.34;APRGLL,0.34;LPFDHH,0.33;LVAPRG,0.32;CWRISD,0.32;GLVAPR,0.3;GKGAFV,0.29;NGKGAF,0.29;SGSGGA,0.29;SRIIDV,0.29;GGAACW,0.28;GAACWR,0.28;ACWRIS,0.26;LTAWAW,0.25;APRGLF,0.24;DHHMLA,0.24;GSGGAA,0.24;LPDPFT,0.24;HSAGAL,0.24;NGKGAL,0.24;AACWRI,0.23;CRRAEI,0.23;SGGAAC,0.23;RIIDAL,0.22;IIDALE,0.22;IVGENV,0.22;NVWFST,0.22;PFPAII,0.22;VGENVW,0.22;GENVWF,0.21;FYDLYG,0.21;PFDHHM,0.21;PRGLLV,0.21;RGQGKF,0.2;AGLVAP,0.2;KGAFVA,0.2;

PPR Group 2

Accession numbers: WP_026076381.1;WP_010258827.1;WP_015546887.1;EKJ91160.1;WP_032848027.1;WP_020530464.1;CCZ96528.1;WP_051915283.1;CDB60728.1;WP_008646530.1;WP_014021959.1;CAZ95028.1;WP_052725648.1;WP_026348373.1;WP_007663944.1;WP_002646813.1;AKP53284.1;WP_044533301.1;WP_025831474.1;WP_005645841.1;KPL12949.1;CDD13403.1;WP_026814534.1;KPK08871.1;CUN53423.1;WP_052362141.1;EIY86435.1;WP_025762030.1;WP_029902766.1;WP_032847076.1;WP_052516303.1;WP_005634913.1;WP_053061258.1;KPJ73319.1;WP_026810456.1;WP_015807334.1;WP_026327150.1;WP_049782645.1;WP_012372945.1;KPK34858.1;WP_013765809.1;CEA16204.1;WP_028523937.1;WP_045029679.1;WP_051892241.1;WP_052673281.1;WP_053181282.1;WP_014218346.1;WP_013565581.1;WP_008155241.1;WP_008150658.1;GAP67958.1;WP_010854535.1;WP_046579001.1;WP_009280786.1;WP_052328999.1;AEW47971.1;WP_009280260.1;WP_007336213.1;EMB14724.1;WP_013926819.1;KLU04072.1;WP_007326352.1;WP_011119680.1;WP_026462043.1;WP_011683513.1;WP_008671480.1;KRO52297.1;KRO32479.1;WP_002655584.1;WP_008512324.1;WP_047488567.1;EPR68257.1;ALJ01753.1;WP_050031137.1;WP_028523679.1;WP_038162569.1;KPK35299.1;WP_020600364.1;WP_044133829.1;EKJ99202.1;WP_020892208.1;AGH13541.1;WP_005634514.1;ETN95190.1;WP_044251066.1;WP_051413508.1;WP_025290401.1;WP_035615147.1;WP_040009599.1;WP_013929002.1;WP_020722596.1;WP_028665868.1;WP_013923691.1;WP_028526496.1;WP_015812586.1;WP_035361769.1;WP_052507937.1;AJP74544.1;WP_015815142.1;AHG93140.1;ACB75560.1;ADB42503.1;WP_008564663.1;WP_020577527.1;WP_015882770.1;WP_026392643.1;WP_025830113.1;WP_020716161.1;WP_019944385.1;WP_007845973.1;WP_016278027.1;WP_007831539.1;CDA96765.1;WP_028664053.1;WP_019990803.1;WP_014264111.1;WP_011685991.1;WP_057279885.1;WP_051023053.1;WP_005841402.1;WP_016270879.1;CDW92038.1;WP_008202249.1;WP_008703720.1;WP_035350050.1;WP_051394862.1;CCY36837.1;EOZ95624.1;WP_050807946.1;WP_009183201.1;WP_026388348.1;WP_051315112.1;WP_056282497.1

Conserved peptides: HSRLGK,0.61;GHSRLG,0.61;DPKGEF,0.55;PKGEFL,0.54;SGCGGA,0.51;GGAALS,0.49;GCGGAA,0.49;SRLGKT,0.46;LIAPRP,0.44;CGGAAL,0.44;GKAALW,0.42;ADPKGE,0.41;WADPKG,0.4;FPHWFC,0.39;KAALWA,0.39;ALWAGA,0.38;WAWGLS,0.37;ALIAPR,0.37;LPVDQH,0.34;GDIDPD,0.34;AWGLSR,0.33;AWAWGL,0.32;KGEFLS,0.32;AALWAG,0.32;AALSRR,0.31;GAALSR,0.31;SRLGKA,0.3;APRPVY,0.29;GYGLAT,0.29;AAWAWG,0.29;APRPLY,0.28;LYVASA,0.28;PRPLYV,0.26;RLGKAA,0.26;RRPELL,0.25;SGEGGA,0.25;PLYVAS,0.25;RPLYVA,0.25;GAWAWG,0.24;ISNDSG,0.24;LGKAAL,0.24;SNDSGC,0.23;IAAWAW,0.23;VYIASA,0.23;LPFDQH,0.22;DSGCGG,0.22;PVYIAS,0.22;NDSGCG,0.22;DPDFDD,0.22;PRPVYI,0.22;RPVYIA,0.22;GEGGAA,0.22;SRAMDY,0.22;VISNDS,0.22;ISNNSG,0.21;IAPRPL,0.21;WAGATD,0.21;IGAWAW,0.2;LWAGAT,0.2;EGGAAL,0.2;WGLSRA,0.2;

PPR Group 3

Accession numbers: WP_026463950.1;ALI97699.1;ABJ83653.1;AHM62667.1;ADB37762.1;WP_046375486.1;WP_013928279.1;WP_052313418.1;WP_018619139.1;WP_054280936.1;AHG92062.1;AFL90432.1;WP_020605401.1;WP_015029583.1;WP_051081028.1;AHF25435.1;WP_040626038.1;WP_020599600.1;WP_008505837.1;WP_009283474.1;AGU12221.1;WP_020714742.1;EDY84748.1;WP_055148089.1;WP_013444200.1;WP_023455433.1;WP_035018484.1;WP_006273025.1;WP_052555620.1;WP_052200844.1;WP_015333415.1;WP_013766809.1;WP_025225517.1;AIE85929.1;WP_036386289.1;WP_056463639.1;WP_056447294.1;WP_035045130.1;WP_031499121.1;WP_050770459.1;WP_019986659.1;WP_013072441.1;WP_008197998.1;KPK85299.1;WP_041840366.1;WP_026950812.1;WP_028522896.1;WP_050058042.1;WP_045827131.1;WP_056048414.1;AIA88983.1;WP_018275724.1;WP_028884865.1;WP_019603900.1;WP_026225096.1;WP_028881720.1;WP_044936167.1;WP_051881570.1;GAP70346.1;WP_028668563.1;WP_052508090.1;WP_029033800.1;WP_031443036.1;WP_026160466.1;WP_015818687.1;WP_026337433.1;WP_028876207.1;WP_035455482.1;WP_047408703.1;WP_037317018.1;WP_028056600.1;WP_008991271.1;EMB17801.1;WP_046368292.1;WP_041523867.1;AGA78907.1;WP_012845124.1;WP_045860974.1;WP_014068100.1;WP_043521012.1;EQB31719.1;WP_012377126.1;ADQ41707.1;WP_051565133.1;CCX55358.1;WP_007079684.1

Conserved peptides: GKAALV,0.73;SRYGKA,0.72;ALCAPR,0.72;LRAWAW,0.7;RYGKAA,0.65;WGALRA,0.63;GALRAW,0.63;AWAWGA,0.62;RAWAWG,0.57;YGKAAL,0.56;LCAPRP,0.56;DWGALR,0.53;HWMAGN,0.52;QADNGA,0.51;YHWMAG,0.51;DNGAGL,0.51;ALRAWA,0.5;ADNGAG,0.5;WMAGNF,0.49;EYHWMA,0.48;GIIGLV,0.45;IIGLVN,0.45;LPVDAH,0.44;DLPVDA,0.44;WAWGAS,0.44;IALCAP,0.42;SSGEGG,0.41;AWGASR,0.41;LIALCA,0.41;RRNFGE,0.4;GVSRYG,0.4;VSRYGK,0.4;KAALVT,0.38;IGLVNK,0.38;EGVSRY,0.38;GLVNKG,0.38;IEGVSR,0.37;GEYHWM,0.37;SGEGGA,0.36;LAWRQH,0.36;GIEGVS,0.36;GEGGAK,0.36;KAALVA,0.35;WGASRA,0.35;VGIEGV,0.35;LIGSSG,0.35;EGGAKL,0.34;HELIAL,0.34;DDWGAL,0.34;CAPRPV,0.33;QHDGGH,0.31;GASRAL,0.31;ASRALD,0.31;RQHDGG,0.31;NGAGLT,0.31;IQADNG,0.31;RKPDDW,0.31;KPDDWG,0.31;NKGQPR,0.31;SIQADN,0.31;LDYLET,0.3;SGEYHW,0.3;GHVDNS,0.29;AWRQHD,0.29;WRQHDG,0.29;

PPR Group 4

Accession numbers: EES67198.1;WP_055269091.1;WP_055299849.1;WP_048696311.1;WP_055220611.1;WP_008762556.1;WP_054960235.1;WP_008649470.1;WP_004299624.1;WP_008642909.1;WP_052319822.1;WP_004305145.1;WP_008776505.1;WP_008024331.1;WP_008998008.1;WP_049701711.1;WP_032847263.1;EKJ90473.1;WP_032848060.1;CDE80374.1;KPK34442.1;WP_041935190.1;AEL27077.1;KLU03021.1;WP_047816142.1;WP_002648538.1;AKP52589.1;WP_041387388.1;WP_052560942.1;WP_052640156.1;WP_040765962.1;EMI20458.1;WP_026903085.1;KPK96875.1;WP_021070011.1;EMI27239.1;WP_008656025.1;WP_007326901.1;WP_011119142.1;EON77291.1

Conserved peptides: KWAMFA,0.95;YEPETA,0.85;EPETAI,0.8;RGFVTL,0.8;PETAIG,0.78;NYWEPW,0.78;PRPFLV,0.78;WEPWYL,0.78;YWEPWY,0.78;GFVTLS,0.78;EPWYLG,0.78;GKWAMF,0.75;FLVSGG,0.75;MFASCL,0.75;AMFASC,0.75;PFLVSG,0.75;WAMFAS,0.75;RPFLVS,0.75;GGKWAM,0.75;APRPFL,0.73;DLHELH,0.7;PWYLGY,0.68;GIVFDE,0.68;DPGIVF,0.63;LAYAAA,0.63;PGIVFD,0.63;FYEPET,0.58;INYWEP,0.58;AAANAW,0.58;TLSIGT,0.58;KRGFVT,0.58;YAAANA,0.58;AYAAAN,0.58;RDFAYQ,0.55;FASCLY,0.55;DFAYQL,0.55;FVTLSI,0.53;VTLSIG,0.53;VFDETK,0.5;WYLGYY,0.5;AYQLTK,0.5;LSIGTT,0.5;KTYSLY,0.5;FAYQLT,0.5;IVFDET,0.5;QLTKRG,0.48;GYYPPP,0.48;AWSDPG,0.48;HSYGGK,0.48;SCLYEK,0.48;LMAPRP,0.48;YLGYYP,0.48;LGYYPP,0.48;MAPRPF,0.48;YGGKWA,0.48;SYGGKW,0.48;YYPPPW,0.48;CLYEKF,0.48;ASCLYE,0.48;LYEKFA,0.48;YINYWE,0.48;GHSYGG,0.48;WSDPGI,0.48;LTKRGF,0.48;

PPR Group 5

Accession numbers: WP_044269389.1;CDB71655.1;WP_026366561.1;WP_007210765.1;WP_007216958.1;WP_029426051.1;WP_009129587.1;CDD95902.1;EEC54465.1;EFV29552.1;CCY54990.1;WP_008103007.1;EMB16514.1;EMI26865.1;WP_018667374.1;WP_045027069.1;CDA48937.1;WP_037251231.1;WP_038555024.1;ADD61496.1;WP_024994021.1;WP_053183329.1;WP_024997057.1;WP_036880740.1;ABG58510.1;CCX56079.1;WP_026475091.1;WP_033150407.1;WP_051651838.1;WP_028911237.1;WP_013065393.1;AHJ97958.1;WP_028906830.1;WP_051522908.1;WP_049961224.1;WP_036913392.1;WP_020714233.1;WP_051313187.1

Conserved peptides: AGKMAL,0.97;GAFDER,0.92;LTIAQE,0.89;ALTIAQ,0.87;GKMALF,0.76;AFDERI,0.76;FDERIA,0.74;PRALLV,0.74;DERIAL,0.74;ALLVLG,0.71;APRALL,0.71;LLVLGN,0.71;ERIALT,0.71;RALLVL,0.71;IALTIA,0.68;PEVEAF,0.68;RIALTI,0.68;WRVSET,0.66;AWRVSE,0.66;EPGGGG,0.66;DKFLLG,0.66;SRLIDG,0.66;QEPGGG,0.63;GGGGAA,0.63;RVSETL,0.63;AQEPGG,0.63;AGAFDE,0.63;IAQEPG,0.63;GCSFAG,0.61;ALFAGA,0.61;MALFAG,0.61;FAGAFD,0.61;GGAAAW,0.61;SFAGKM,0.61;CSFAGK,0.61;GAAAWR,0.61;FAGKMA,0.61;KMALFA,0.61;GGGAAA,0.61;LFAGAF,0.61;TIAQEP,0.61;VSETLG,0.58;DGSGRS,0.55;LGNPDY,0.53;GSGRST,0.53;ETLGRT,0.5;YPEVEA,0.5;QYPEVE,0.5;VETLGR,0.5;GNEPIN,0.5;RGNEPI,0.5;RLIDGL,0.5;LVLGNP,0.47;LIDGLE,0.47;QKRGNE,0.45;DESGYV,0.45;AAAWRV,0.45;WLADES,0.45;AAWRVS,0.45;FGIADR,0.45;SGCSFA,0.45;ESGYVS,0.45;PGGGGA,0.45;

PPR Group 6

Accession numbers: ALL79991.1;WP_010243407.1;WP_050802290.1;ALE76107.1;ALL78758.1;ALE77052.1;ALE86700.1;ALL85951.1;ALE77971.1;ALE83935.1;WP_031530548.1;WP_026628565.1;WP_056291684.1;WP_020597689.1;WP_019940230.1;WP_015812497.1;WP_018621258.1;WP_051737618.1;WP_016197406.1;WP_020892374.1;WP_014022617.1;WP_048643867.1;WP_010853535.1;WP_038164644.1;WP_050025049.1;WP_041047781.1;WP_020626384.1;WP_020617403.1;WP_051350394.1;WP_009511085.1;WP_008708734.1;WP_014270200.1;WP_051584756.1;WP_052574025.1;WP_006978972.1

Conserved peptides: HSRGGK,0.86;GHSRGG,0.86;AWAWGA,0.77;LPVDQH,0.77;WAWGAS,0.63;SRGGKA,0.63;AWGASR,0.63;VIGHSR,0.46;AVIGHS,0.46;VDQHML,0.46;VGHSRG,0.46;PVDQHM,0.46;WGASRA,0.43;SGSTGA,0.4;VVGHSR,0.4;ALWAGA,0.4;DLWADP,0.37;GKAALW,0.37;WAGAQD,0.37;LWAGAQ,0.37;GAWAWG,0.37;GGKASL,0.34;NSGSTG,0.34;NNSGST,0.34;DQHMLI,0.34;MSYHLR,0.34;IGHSRG,0.34;GKASLW,0.34;RGGKAS,0.34;VAVIGH,0.31;SYHLRS,0.31;FPHWFP,0.31;PVFLLI,0.31;WGASRV,0.31;YHLRSG,0.31;QHMLIA,0.29;ISNNSG,0.29;GAQDTR,0.29;DHRGSV,0.29;IGAWAW,0.29;GSTGAK,0.29;FPHWFN,0.29;IDHRGS,0.29;VFLLIN,0.29;SGGHGL,0.29;RGGKAA,0.29;RSGGHG,0.29;AGYAFA,0.29;

PPR Group 7

Accession numbers: WP_014021405.1;WP_048642817.1;WP_040415030.1;WP_019208176.1;EPT34705.1;WP_018616961.1;WP_015245027.1;WP_012780519.1;WP_010853638.1;WP_036660677.1;WP_009595468.1;WP_046680731.1;WP_015735646.1;WP_010584327.1;KLU05545.1;WP_054955593.1;WP_054404517.1;WP_052549780.1;WP_008583533.1;WP_053490367.1;WP_002654417.1;WP_020475080.1;WP_008703722.1

Conserved peptides: IGHSLG,1.0;GHSLGG,1.0;VIGHSL,0.78;GVIGHS,0.74;IGVIGH,0.74;SLGGHN,0.7;HSLGGH,0.7;RIGVIG,0.65;LGGHNA,0.48;VVSSCG,0.48;PDYPSF,0.43;YVVIAP,0.43;VSSCGF,0.43;RGYVVI,0.43;DYPSFG,0.43;GYVVIA,0.43;AVVSSC,0.43;KAVVSS,0.43;VIAPDY,0.39;IAPDYP,0.39;VVIAPD,0.39;LGPWAQ,0.39;IKAVVS,0.35;RLGPWA,0.35;APDYPS,0.35;RIKAVV,0.35;ELAERG,0.35;GGRLGP,0.35;GRLGPW,0.35;AERGYV,0.35;ERGYVV,0.35;LAERGY,0.35;QDRYMP,0.3;SLGGYN,0.3;AQDRYM,0.3;WAQDRY,0.3;YGGRLG,0.3;REWFSH,0.3;GPWAQD,0.3;PWAQDR,0.3;HSLGGY,0.3;YPSFGE,0.26;EWFSHI,0.26;WFSHIP,0.26;GHDFPP,0.26;SSCGFT,0.26;

PPR Group 8

Accession numbers: XP_003352714.1;XP_001221315.1;XP_003656155.1;KOP49092.1;KKY31057.1;XP_003665710.1;XP_001912192.1;CDP22312.1;XP_007840382.1;GAP88126.1;XP_008076478.1;XP_007919238.1;KJR87236.1;XP_007796291.1;KIH94860.1;KNG51094.1;XP_003833939.1;KEZ45973.1;XP_001838356.2;ERS96496.1;XP_007348092.1;KDQ18272.1

Conserved peptides: VTGCSR,0.95;CSRLGK,0.95;GCSRLG,0.95;TGCSRL,0.91;GVTGCS,0.91;TAWAWG,0.91;LTAWAW,0.91;GVLTAW,0.86;VLTAWA,0.86;IGVLTA,0.86;ALAAGL,0.86;WAWGFH,0.82;DIGVLT,0.82;AWAWGF,0.82;AWGFHR,0.82;MPMSSG,0.77;AAIAPR,0.77;CRQPEI,0.77;MSSGVQ,0.73;TMPMSS,0.73;HCDMSG,0.73;AIAPRA,0.73;PMSSGV,0.73;SSGVQG,0.73;INIGGM,0.68;GGHCDM,0.68;VGVTGC,0.68;GHCDMS,0.68;DQGTGD,0.64;RDIGVL,0.64;GYYPDH,0.64;GRDIGV,0.64;IDQGTG,0.64;SGGHCD,0.64;SGQGEN,0.59;HRTLDA,0.59;AAGLFD,0.59;QEYQYG,0.59;LAAGLF,0.59;AAAIAP,0.59;GQGENL,0.59;LTMPMS,0.59;NIGGMQ,0.59;WGFHRT,0.59;LQEYQY,0.59;RVGVTG,0.59;FHRTLD,0.59;RSGGHC,0.59;LPYDAH,0.59;GFHRTL,0.59;VYDWLG,0.55;YGYYPD,0.55;IAPRAL,0.55;TVPEID,0.55;HTIAAA,0.55;GENLEN,0.55;QYGYYP,0.55;IAAAIA,0.55;QGENLE,0.55;YQYGYY,0.55;AHTIAA,0.55;PYDAHT,0.55;EYQYGY,0.55;VINIGG,0.55;

PPR Group 9

Accession numbers: WP_007337469.1;WP_008665192.1;WP_008661262.1;WP_007329823.1;WP_007332089.1;WP_011120539.1;WP_047816899.1;WP_008695296.1;WP_012913141.1;ADY59785.1;WP_052299426.1;WP_002646926.1;WP_002650217.1;WP_007416434.1;WP_006981556.1;WP_020470185.1;KPK73794.1;WP_050030361.1;WP_051946928.1;WP_020469077.1;WP_009964612.1

Conserved peptides: SGGGAY,0.9;GGGAYS,0.9;GRSGGG,0.9;GGAYSW,0.9;RSGGGA,0.9;GHCDCM,0.86;RGYTPA,0.81;GYTPAG,0.81;TPAGVE,0.81;TGRSGG,0.76;YTPAGV,0.76;LYVCGH,0.71;PGLYVT,0.71;GVEAWN,0.67;PAGVEA,0.67;AGVEAW,0.67;NGYVCL,0.67;VTGRSG,0.62;GVTGRS,0.62;HHGTYR,0.57;ALVAPR,0.57;NHVVDG,0.57;FARNGY,0.57;VVDGCV,0.57;APRPLL,0.57;IGVTGR,0.57;ARNGYV,0.57;YVCGHG,0.57;HCDCMF,0.57;RNGYVC,0.57;HVVDGC,0.57;WFARNG,0.57;AALVAP,0.57;RIGVTG,0.52;LYVTAN,0.52;TIQLGE,0.52;YVTANL,0.52;DTIQLG,0.52;IDTIQL,0.52;VTANLY,0.52;DSIFPL,0.52;VLYVCG,0.48;MPGLYV,0.48;SMPGLY,0.48;TANLYR,0.48;HGTYRE,0.48;PRPLLI,0.48;NRGYTP,0.48;NLYRPA,0.48;ANLYRP,0.48;AVPVAG,0.48;GAYSWW,0.48;

PPR Group 10

Accession numbers: WP_034769177.1;WP_035580241.1;WP_051596017.1;WP_034797934.1;KCZ52458.1;WP_051624504.1;WP_051614960.1;WP_034812634.1;WP_051618700.1;WP_035551120.1;ABI77990.1;WP_018148598.1;WP_051612534.1;WP_022699599.1

Conserved peptides: DVWSDP,0.93;RDVWSD,0.93;RRDVWS,0.93;LLGNGR,0.86;WSDPNS,0.86;LGNGRR,0.86;GRRDVW,0.86;NGRRDV,0.86;GNGRRD,0.86;VWSDPN,0.86;VLLGNG,0.79;LRPGGH,0.79;RPGGHS,0.79;PVLLGN,0.71;LMAWAY,0.71;TPVLLG,0.71;APTPVL,0.71;PTPVLL,0.71;AYASFY,0.71;LYGPWP,0.71;AHQSGF,0.64;QSGFAG,0.64;RGTLEE,0.64;HQSGFA,0.64;SGFAGA,0.64;GRGTLE,0.64;VMGHSR,0.64;QHELLA,0.57;DQHELL,0.57;HELLAL,0.57;SDPNSS,0.57;YPHWLA,0.57;PVDQHE,0.57;VDQHEL,0.57;WLRPGG,0.57;LPVDQH,0.57;PHWLAP,0.57;GGHSIV,0.57;GASLSR,0.5;MGHSRH,0.5;ASLSRS,0.5;NSSFRA,0.5;LAYASF,0.5;AGASLS,0.5;LLAPTP,0.5;GHSRHG,0.5;HSRHGK,0.5;PNSSFR,0.5;LAPTPV,0.5;GFAGAS,0.5;PGGHSI,0.5;DPNSSF,0.5;TLMAWA,0.5;FAGASL,0.5;SSFRAA,0.5;GLAYAS,0.5;

PPR Group 11

Accession numbers: WP_014114841.1;CUB35778.1;WP_010331548.1;WP_010789557.1;WP_024122555.1;WP_010329545.1;WP_019259230.1;WP_003237986.1;WP_044153757.1;WP_039074807.1;WP_042201223.1

Conserved peptides: IGMSMG,1.0;GLMAWW,1.0;GTIGMS,1.0;MSMGGL,1.0;MGGLMA,1.0;GMSMGG,1.0;TIGMSM,1.0;IGTIGM,1.0;SMGGLM,1.0;GGLMAW,1.0;IAPRPH,0.91;SGHFET,0.91;RSASGH,0.91;GHFETA,0.91;RPHLSL,0.91;AKHFSA,0.91;WGFGDR,0.91;APRPHL,0.91;KHFSAS,0.91;RVMWGM,0.91;DHWGFG,0.91;FKEMLL,0.91;CSQVDH,0.91;ASGHFE,0.91;LAKHFS,0.91;PRPHLS,0.91;HWGFGD,0.91;FGDRRG,0.91;RIGTIG,0.91;SASGHF,0.91;VMWGMM,0.91;GFGDRR,0.91;GRVMWG,0.91;GDRRGK,0.91;

PPR Group 12

Accession numbers: CAB55348.1;WP_026053007.1;WP_019679655.1;WP_037301098.1;WP_028516954.1;WP_024861904.1;WP_009985705.1;WP_037298323.1;WP_028514285.1

Conserved peptides: PDPFIF,1.0;MDGSKV,1.0;FIFMDG,1.0;MYEYYM,1.0;ISCMYE,1.0;YYMYGK,1.0;GKTASF,1.0;RKSTGK,1.0;EGGAPV,1.0;EYYMYG,1.0;KSTGKT,1.0;EISCMY,1.0;FMDGSK,1.0;VRHEGG,1.0;HEGGAP,1.0;TGKTAS,1.0;DGSDDE,1.0;CMYEYY,1.0;GSKVES,1.0;PFIFMD,1.0;GAAKEL,1.0;ELNINP,1.0;IFMDGS,1.0;DWWKRQ,1.0;YEYYMY,1.0;RHEGGA,1.0;KRKSTG,1.0;STGKTA,1.0;KELNIN,1.0;GGAPVI,1.0;YMYGKW,1.0;SCMYEY,1.0;DGSKVE,1.0;DPFIFM,1.0;AKELNI,1.0;LMAWSW,1.0;MAWSWG,1.0;AAKELN,1.0;

PPR Group 13

Accession numbers: WP_037992621.1;WP_038010261.1;WP_045826467.1;WP_037986797.1;WP_037990893.1;WP_018014275.1;WP_028876482.1;ACR11863.1;WP_051092532.1;WP_018415785.1;WP_053085126.1;WP_044388857.1;CQR60065.1;WP_029386926.1;KMS70116.1;WP_052454436.1;WP_052809350.1;WP_026337924.1;WP_051759442.1;WP_051708916.1;WP_015802876.1;WP_033429312.1;WP_053716409.1;WP_051366042.1;WP_020737434.1;WP_013534398.1

Conserved peptides: VTGCSR,0.96;GAFDQR,0.88;YKALGA,0.85;AFDQRI,0.77;SRFGKG,0.73;RFGKGA,0.73;GCSRFG,0.73;CSRFGK,0.73;TGCSRF,0.69;FGKGAF,0.69;SAYGEQ,0.65;VAPRGL,0.62;SSAYGE,0.58;VAALGG,0.58;AALGGA,0.58;IYKALG,0.58;GAEIYK,0.58;ALGGAE,0.58;EIYKAL,0.58;AEIYKA,0.58;MVAPRG,0.58;AGAFDQ,0.58;GVTGCS,0.58;APRGLL,0.54;WLGDAF,0.54;LTMPIE,0.5;PGEGAQ,0.5;KQGAFY,0.5;GEGAQS,0.5;RLIDVI,0.5;TMPIES,0.5;MPIESG,0.5;GPFPAV,0.5;SRLIDV,0.5;DGTHCA,0.5;FYDIYG,0.46;DWRCRR,0.46;YDIYGS,0.46;FDQRID,0.46;CRRAEI,0.46;DQRIDL,0.46;LIDVIE,0.46;AYGEQP,0.46;GAFYDI,0.46;GMVAPR,0.46;PWLGDA,0.46;VGMVAP,0.46;YGEQPW,0.46;AFYDIY,0.46;QRIDLT,0.46;

PPR Group 14

Accession numbers: WP_038133810.1;WP_019112243.1;WP_013928155.1;WP_044240939.1;ADB41587.1;WP_053989841.1;WP_008097689.1;KPL24379.1;WP_052573519.1

Conserved peptides: SAHFFG,0.89;VSAHFF,0.89;SGGGTQ,0.89;HFFGGC,0.89;CESGMP,0.89;AHFFGG,0.89;GGGTQT,0.67;EKHDYG,0.56;GGTQTF,0.56;SGMPIH,0.56;MVSAHF,0.56;APRPQL,0.56;ESGMPI,0.56;VMVSAH,0.56;VCESGM,0.44;SIRALD,0.44;LQTWNS,0.44;NSIRAL,0.44;FGGCVC,0.44;GGCVCE,0.44;FFGGCV,0.44;TNNVEI,0.44;VENVHL,0.44;WNSIRA,0.44;TQTFIL,0.44;ESGGGT,0.44;AAPRPQ,0.44;CVCESG,0.44;GCVCES,0.44;GMPIHK,0.44;VPVVMV,0.44;PVVMVS,0.44;ENVHLP,0.44;GESGGG,0.44;NVEIAA,0.33;LAAPRP,0.33;PQLLIS,0.33;NNVEIA,0.33;YGPSKR,0.33;VVMVSA,0.33;EGHDYG,0.33;IAVSVP,0.33;ALAAPR,0.33;HDYGPS,0.33;RAVDFL,0.33;RIAVSV,0.33;GTQTFI,0.33;AALAAP,0.33;SVPVVM,0.33;TWNSIR,0.33;ILAAID,0.33;DYGPSK,0.33;TGESGG,0.33;QTWNSI,0.33;RIGMTG,0.33;SGMPVH,0.33;QTFILA,0.33;FILAAI,0.33;QTNNVE,0.33;PRPQLL,0.33;ESGMPV,0.33;QVSAHF,0.33;TFILAA,0.33;LLVSDG,0.33;FLAKHL,0.33;

PPR Group 15

Accession numbers: WP_020472689.1;WP_015250164.1;WP_010582671.1;WP_010050401.1;WP_052561012.1;WP_007415361.1;WP_012910898.1;WP_006980718.1

Conserved peptides: RRPELK,0.75;QAGCGG,0.75;CAPRPV,0.75;APRPVL,0.75;GKTALL,0.63;RPGKHS,0.63;KTALLA,0.63;GCGGTA,0.63;FPHWFC,0.63;VVGHSR,0.63;CGGTAP,0.63;PGKHSM,0.63;KRRPEL,0.63;AGCGGT,0.63;AVVGHS,0.63;YAVATF,0.5;HSRLGK,0.5;AFDDRI,0.5;AICAPR,0.5;LPFDQH,0.5;GHSRLG,0.5;GGTAPS,0.5;DTWANP,0.5;GTAPSR,0.5;VGHSRL,0.5;ELKALF,0.5;QHYMYG,0.5;GYAVAT,0.5;IAVVGH,0.5;FQHYMY,0.5;EKRRPE,0.5;FDDRIA,0.5;LFQHYM,0.5;PRPVLF,0.5;GQFEVL,0.5;AVATFY,0.5;RPELKA,0.5;PELKAL,0.5;ALFQHY,0.5;NFCGNH,0.5;

PPR Group 16

Accession numbers: WP_045091931.1;WP_025486811.1;WP_008717200.1;WP_007860043.1;WP_026891673.1;WP_024838285.1;WP_008721239.1

Conserved peptides: DMDEYD,1.0;RFAMAV,1.0;GKTALW,1.0;HDMDEY,1.0;DEYDWN,1.0;DSWADP,1.0;AWAASR,1.0;GNSGDA,1.0;TIISRG,1.0;SRGYAC,1.0;PAETII,1.0;MDEYDW,1.0;RGGKTA,1.0;AETIIS,1.0;AMAVSS,1.0;GGKTAL,1.0;HSRGGK,1.0;TVEVEA,1.0;FAMAVS,1.0;YPAETI,1.0;ISRGYA,1.0;RGYACA,1.0;IISRGY,1.0;FYPAET,1.0;LIDEKR,1.0;SRGGKT,1.0;ETIISR,1.0;FRTQEV,1.0;WAWAAS,1.0;GHSRGG,1.0;MAVSSC,1.0;PLIDEK,1.0;QDERFA,0.86;AQDERF,0.86;DERFAM,0.86;ERFAMA,0.86;VGHSRG,0.86;

PPR Group 17

Accession numbers: WP_014019951.1;WP_020893432.1;WP_048641606.1;WP_010853637.1;WP_019540171.1;WP_017260061.1;WP_018616917.1

Conserved peptides: GHSRNG,1.0;HSRNGK,1.0;AFDERI,0.86;AAFDER,0.86;TFPDPP,0.71;SGTFPD,0.71;RNGKQS,0.71;HPRLRF,0.71;SRNGKQ,0.71;NGKQSL,0.71;AAAFDE,0.71;GEGPFP,0.71;IAVRRG,0.71;AVRRGY,0.71;TGHSRN,0.71;PIDQNS,0.71;GPFPVF,0.71;GTFPDP,0.71;QIAVRR,0.71;DYLYTL,0.71;EGPFPV,0.71;GADAKD,0.57;YPDYDW,0.57;AGADAK,0.57;RRGYMG,0.57;RYTDER,0.57;SLMALI,0.57;PWAIEQ,0.57;QWNHRG,0.57;DPWAIE,0.57;IAPNAL,0.57;NSLMAL,0.57;YRYTDE,0.57;SSGGTG,0.57;AKLTLE,0.57;VRRGYM,0.57;PFPVFM,0.57;IELRFG,0.57;GDPWAI,0.57;LIAPNA,0.57;QNSLMA,0.57;PYRYTD,0.57;ADAKDD,0.57;PVFMTQ,0.57;WLDIQF,0.57;GGDPWA,0.57;ALIAPN,0.57;DQNSLM,0.57;PPGEGP,0.57;YAGADA,0.57;DAKDDT,0.57;KAKLTL,0.57;ITGHSR,0.57;ITSSGG,0.57;DWLDIQ,0.57;FDERIT,0.57;GGGDPW,0.57;MALIAP,0.57;TSSGGT,0.57;WNHRGW,0.57;IYAGAD,0.57;VDYLYT,0.57;YTDERH,0.57;HKAKLT,0.57;LMALIA,0.57;SSSIRE,0.57;DERITA,0.57;PGEGPF,0.57;IDQNSL,0.57;

PPR Group 18

Accession numbers: XP_001832002.2;XP_001832002.2;XP_001832002.2;XP_001832002.2;XP_001832002.2;XP_001832002.2

Conserved peptides: LVAGAF,1.0;NKFLRG,1.0;PGQQYP,1.0;HDHCVF,1.0;VSIVGG,1.0;APRGLL,1.0;ALVAGA,1.0;FPGQQY,1.0;GHDHCV,1.0;SIVGGH,1.0;DHCVFP,1.0;GQQYPE,1.0;CRRQEI,1.0;GVSIVG,1.0;VAPRGL,1.0;VFPGQQ,1.0;GGHDHC,1.0;LGVSIV,1.0;GAGCWR,1.0;CVFPGQ,1.0;VGGHDH,1.0;RLGVSI,1.0;DRLGVS,1.0;IVGGHD,1.0;HCVFPG,1.0;GGAGCW,1.0;

PPR Group 19

Accession numbers: WP_018618177.1;WP_020605952.1;WP_035429986.1;ESQ83290.1;WP_052201010.1;WP_014784786.1

Conserved peptides: ALVAPR,1.0;LVAPRP,1.0;WGMSRV,0.67;DPKGEF,0.67;DAHELI,0.67;VAAGPV,0.67;ALWAGA,0.67;AHELIA,0.67;YFETDK,0.67;IALVAP,0.67;VDAHEL,0.67;DYFETD,0.67;LIALVA,0.67;VAPRPV,0.67;PKGEFL,0.67;ELIALV,0.67;HELIAL,0.67;PVDAHE,0.67;WAWGMS,0.5;AWGMSR,0.5;KGEFLA,0.5;FETDKA,0.5;AAGPVY,0.5;

PPR Group 20

Accession numbers: WP_002653778.1;WP_010583615.1;WP_013628962.1;WP_012910589.1;WP_015245406.1;ADI22909.1

Conserved peptides: HSLGAK,1.0;GHSLGA,1.0;SLGAKE,1.0;APWYLG,0.83;APRPFL,0.67;NWEAPW,0.5;LVLGGE,0.5;IAPRPF,0.5;AFDDRI,0.5;YLAAFD,0.5;FLVLGG,0.5;LYLTAF,0.5;LIAPRP,0.5;TAFDDR,0.5;YLTAFD,0.5;SSEGGI,0.5;WEAPWY,0.5;RPFLVL,0.5;PFLVLG,0.5;SEGGIG,0.5;EAPWYL,0.5;LGAKEV,0.5;LTAFDD,0.5;VDPQRI,0.33;KEVLYL,0.33;PWYLGP,0.33;AKEVLY,0.33;VSSEGG,0.33;FLLIGG,0.33;PWYLGE,0.33;IGHSLG,0.33;PQRIGA,0.33;ISSEGG,0.33;RLGLLN,0.33;GAKEVL,0.33;AIGHSL,0.33;DPQRIG,0.33;LGLLNH,0.33;VLGGES,0.33;PRPFLL,0.33;PRPFLV,0.33;LGGESG,0.33;AVGHSL,0.33;SNWEAP,0.33;TISSEG,0.33;VGHSLG,0.33;PRNFLW,0.33;ALIAPR,0.33;LYLAAF,0.33;RAVDVL,0.33;AYGHSL,0.33;VASEGG,0.33;AVDVLL,0.33;YGHSLG,0.33;NHGQGH,0.33;RIGAYG,0.33;DGERSW,0.33;IGAYGH,0.33;AAVASE,0.33;GERSWP,0.33;GAYGHS,0.33;AVASEG,0.33;

PPR Group 21

Accession numbers: XP_010493832.1;XP_010454914.1;KFK27821.1;XP_002874282.1;XP_006286737.1;XP_013611518.1;XP_013737468.1;BAH20299.1;NP_974835.1;NP_001078622.1;XP_013660859.1;XP_006394826.1;XP_010454911.1;XP_010454912.1;XP_013620804.1;XP_013616728.1;XP_013699980.1;XP_013699976.1;XP_013711081.1;XP_004501179.1;XP_010541651.1;XP_009129948.1;XP_012077417.1;XP_010249673.1;KCW74784.1;XP_010491458.1;XP_010057593.1;XP_007223085.1;KDP34193.1;XP_009388066.1;XP_006476074.1;KDO79751.1

Conserved peptides: DLIKLA,1.0;QGFRWA,0.97;GFRWAI,0.97;WDLIKL,0.97;AWFAAA,0.91;VQGFRW,0.91;RIGITG,0.91;GVQGFR,0.88;VWDLIK,0.88;IGVQGF,0.88;IAPRPL,0.88;DTVWDL,0.84;PFIFDT,0.84;TVWDLI,0.84;YHGERA,0.81;RYHGER,0.81;TMPFIF,0.81;DSRYHG,0.81;MPFIFD,0.81;SRYHGE,0.81;IDKEVV,0.78;FDTVWD,0.78;RIAPGL,0.78;IFDTVW,0.78;WFDKFL,0.78;FIFDTV,0.78;GGMHAW,0.75;SDWFDK,0.75;LGGMHA,0.75;APGLAS,0.75;DPRCPL,0.75;IAPGLA,0.75;DWFDKF,0.75;SLGGMH,0.75;GMHAWF,0.72;DTRYSV,0.72;AADTRY,0.69;HAWFAA,0.69;FDKFLK,0.69;GITGIS,0.69;AAADTR,0.69;FAAAAD,0.69;WFAAAA,0.69;LIGVQG,0.69;PLIGVQ,0.69;IGITGI,0.69;APRPLY,0.69;MHAWFA,0.69;FRWAID,0.66;ITGISL,0.66;ASRGYV,0.66;TGISLG,0.66;GISLGG,0.66;ISLGGM,0.66;SRGYVA,0.66;IKLAEY,0.63;LIKLAE,0.63;AAAADT,0.63;KLAEYL,0.63;RWAIDN,0.63;VPLIGV,0.59;IGLDSR,0.59;EVVEKV,0.59;KESSDW,0.59;ESSDWF,0.59;GLDSRY,0.59;LDSRYH,0.59;RGYVAI,0.59;AIGLDS,0.59;SSDWFD,0.59;

PPR Group 22

Accession numbers: WP_025747041.1;WP_034628798.1;KRG08906.1;WP_012995691.1;WP_041048404.1

Conserved peptides: CMGISG,0.8;GISGGG,0.8;MGISGG,0.8;GCMGIS,0.8;GLIAPR,0.6;NTFKDS,0.6;IAPRPL,0.6;IGLIAP,0.6;ISGGGL,0.6;SGYANT,0.6;APRPLL,0.6;IGCMGI,0.6;SGGGLV,0.6;GYANTF,0.6;LIAPRP,0.4;ATVISG,0.4;TVISGY,0.4;HCLDNY,0.4;LIGLIA,0.4;RRHCLD,0.4;GGGLVC,0.4;KATVIS,0.4;RHCLDN,0.4;DYLLTR,0.4;RIGCMG,0.4;KIGCMG,0.4;IKATVI,0.4;IDERIK,0.4;YANTFK,0.4;AFTSAI,0.4;DERIKA,0.4;ANTFKD,0.4;FTSAID,0.4;VISGYA,0.4;RIKATV,0.4;TSAIDE,0.4;ISGYAN,0.4;TFKDSI,0.4;SAIDER,0.4;FKDSIM,0.4;AIDERI,0.4;AVISGY,0.4;PRPLLI,0.4;RPLLIE,0.4;MSIYHC,0.4;

PPR Group 23

Accession numbers: WP_021634156.1;WP_050018426.1;WP_016360159.1;CUP65187.1;CDE48405.1

Conserved peptides: GKAALL,1.0;YWFCGN,1.0;RLGKAA,1.0;PYWFCG,1.0;LGKAAL,1.0;ALLCGA,1.0;SRLGKA,1.0;FPYWFC,1.0;AALLCG,1.0;HSRLGK,1.0;KAALLC,1.0;GHSRLG,1.0;LRPGTH,0.8;CRNFPY,0.8;GKEGEN,0.8;VADLCR,0.8;NFPYWF,0.8;HLRPGT,0.8;DWKLFM,0.8;AVARVS,0.8;YQDIAP,0.8;LFPGNA,0.8;TEWETV,0.8;RNFPYW,0.8;ENVADL,0.8;KEGENV,0.8;ADLCRN,0.8;NVADLC,0.8;FRGKEG,0.8;SYQDIA,0.8;WKLFME,0.8;LCGAMD,0.8;VARVSY,0.8;QDIAPD,0.8;RGKEGE,0.8;VTPVYE,0.8;ETEWET,0.8;EGENVA,0.8;DLCRNF,0.8;IMDYLV,0.8;RIMDYL,0.8;RPGTHY,0.8;KLFMEY,0.8;GWETEW,0.8;SYPFQL,0.8;ARVSYQ,0.8;DIAPDQ,0.8;SRIMDY,0.8;VSYQDI,0.8;LLCGAM,0.8;LFRGKE,0.8;GENVAD,0.8;LCRNFP,0.8;AFGGKA,0.8;AVTPVY,0.8;LFMEYR,0.8;WETEWE,0.8;NAFGGK,0.8;RVSYQD,0.8;

PPR Group 24

Accession numbers: WP_031498316.1;WP_015250093.1;WP_002648474.1;WP_020717255.1;WP_010586045.1

Conserved peptides: TGNSGG,0.8;GNSGGG,0.8;NSGGGT,0.8;RALDYL,0.6;SGGGTL,0.6;CTGNSG,0.4;IRALDY,0.4;GVTGNS,0.4;GGGTLT,0.4;DGIRAL,0.4;VTGNSG,0.4;DYLVSR,0.4;ALDDRV,0.4;QAAAPV,0.4;LDDRVQ,0.4;AAAPVC,0.4;GCTGNS,0.4;DDRVQA,0.4;RAPKPT,0.4;GIRALD,0.4;DRVQAA,0.4;APKPTL,0.4;SRPEID,0.4;RVQAAA,0.4;MALDDR,0.4;VQAAAP,0.4;RSEVDP,0.4;AALDDR,0.4;SEVDPK,0.4;EVDPKR,0.4;ALDDRI,0.4;IGPQDA,0.4;VAAPSC,0.4;GPQDAE,0.4;AAPSCY,0.4;PQDAEQ,0.4;APSCYI,0.4;PSCYIT,0.4;SCYITS,0.4;
